# Clinical characteristics of invasive pulmonary aspergillosis in patients with COVID-19 in Zhejiang, China: a retrospective case series

**DOI:** 10.1186/s13054-020-03046-7

**Published:** 2020-06-05

**Authors:** Jie Wang, Qing Yang, Piaopiao Zhang, Jifang Sheng, Jianying Zhou, Tingting Qu

**Affiliations:** 1 Respiratory Department, The First Affiliated Hospital, Zhejiang University School of Medicine, Hangzhou, Zhejiang China; 2 State Key Laboratory for Diagnosis and Treatment of Infectious Disease, The First Affiliated Hospital, Zhejiang University School of Medicine, 79# Qingchun East Road, Hangzhou, 310001 Zhejiang China; 3 Infection Control Department, The First Affiliated Hospital, Zhejiang University School of Medicine, Hangzhou, Zhejiang China

**Keywords:** COVID-19, SARS-CoV-2, Invasive pulmonary aspergillosis, Bronchoscopy

To date, COVID-19 has been pandemic across the whole world, whereas around 20% of patients require treatment in intensive care units (ICUs) [1, 2]. However, the clinical characteristics and risk factors of IPA in patients with COVID-19 are not well defined. We collected clinical data for 104 patients with COVID-19 between January and March 2020 in the First Affiliated Hospital of Zhejiang University, China. All patients were diagnosed with COVID-19 by positive PCR results. IPA was defined based on proven or probable criteria according to the revision and update of the European Organization for Research and Treatment of Cancer and the Mycoses Study Group Education and Research Consortium [3]. All the statistical analyses were done with the SPSS software 25.0.

As shown in Table 1, of the 104 patients with COVID-19, 8 (7.7%) patients had IPA diagnosed, with obviously older age than the patients without IPA (73 years ± 13 years vs 53 years ± 15 years; *P* < .001). All the 8 patients were male and had IPA diagnosed after SARS-Cov-2-negative results were obtained. IPA was diagnosed a median of 21 days after the onset of COVID-19 symptoms and a median of 19 days after admission. *Aspergillus* was cultured positive from the sputum or bronchoalveolar lavage fluid (BALF) samples of the 8 patients (4 cases from sputum and 4 cases from BALF), and all the 8 cases were *Aspergillus fumigatus*. Among the 8 patients co-infected with SARS-CoV-2 and *Aspergillus*, 6 patients were administrated with glucocorticoids, 4 patients mechanical ventilation, 1 patient continuous renal replacement therapy (CRRT)-supported, and 1 patient extracorporeal membrane oxygenation (ECMO)-supported before IPA occurrence. There were significant differences in hypertension, COPD, and chronic kidney disease between the *Aspergillus*-positive and *Aspergillus*-negative groups (*P* < .05). Prior to the development of IPA, 50.0% vs 11.5% of patients in the *Aspergillus*-positive and *Aspergillus*-negative groups required mechanical ventilation support, respectively (*P* < .05). Most patients in the 2 groups received glucocorticoids (75.5% vs 59.4%, respectively). There were no significant differences in maximum methylprednisolone equivalent dosage between the 2 groups (methylprednisolone, 40–80 mg/daily). Multivariate analysis showed that older age, initial antibiotic usage of β-lactamase inhibitor combination, mechanical ventilation, and COPD but not hypertension and glucocorticoid therapy were independent risk factors for IPA in patients with COVID-19.

**Table 1:** Clinical features and selected laboratory abnormalities of COVID-19 patients with or without *Aspergillus* infection

Variable	Aspergillus positive (*n* = 8)	Aspergillus negative (*n* = 96)	*P* value
Demographics			
Age, mean (SD), years	73 (13)	53 (15)	< 0.001
Male sex, no. (%)	8 (100)	54 (56.3)	0.02
Underlying disease, no. (%)			
Any	7 (87.5)	36 (37.5)	0.008
Hypertension	7 (87.5)	31 (32.3)	0.003
Diabetes mellitus	2 (25)	11 (11.5)	0.262
Heart disease	1 (12.5)	6 (6.3)	0.439
COPD	2 (25)	2 (2.1)	0.029
Cancer	0 (0)	1 (1.0)	1
Immunodeficiency	0 (0)	0 (0)	–
Chronic kidney disease	2 (25)	0 (0)	0.005
Pregnancy	0 (0)	3 (3.1)	1
Smoking in the past 1 year, no. (%)	2 (25)	6 (6.3)	0.115
Severe/critical type, no. (%)	8 (100)	70 (72.9)	0.196
Complications, no. (%)			
ARDS	4 (50)	38 (39.6)	0.712
Shock	0 (0)	0 (0)	–
Liver damage	1 (12.5)	7 (7.3)	0.485
Acute kidney injury	1 (12.5)	0 (0)	0.077
Treatment, no. (%)			
Mechanical ventilation	4 (50)	11 (11.5)	0.014
ECMO	1 (12.5)	3 (3.1)	0.278
CRRT	1 (12.5)	1 (1.0)	0.149
Corticosteroid treatment, no. (%)	6 (75)	57 (59.4)	0.475
Maximum methylprednisolone equivalent dosage, median (IQR), mg/day	70 (5–80)	40 (0–60)	0.191
Administration of antiviral treatment	8 (100)	89 (92.7)	1
Initial antibiotic treatment, no./total (%)	6 (75)	46 (47.9)	0.269
3rd-generation cephalosporin	0 (0)	7 (7.3)	1
Fluoroquinolone	3 (37.5)	26 (27.1)	0.683
β-Lactamase inhibitors	6 (75)	17 (17.7)	0.001
ICU admission, no./total (%)	8 (100)	18 (18.8)	0

Chest computed tomography (CT) scans of patients with COVID-2019 and IPA are shown in Fig. 1. Among the patients with COVID-2019 and IPA, typical aggressive pneumoconiosis imaging changes were shown in the early phase such as nodules with cavities and dendritic signs. However, in the late phase of the disease, the imaging changes of IPA were atypical, and some lesions might be hidden in the consolidation or interstitial changes. Fibreoptic bronchoscopy showed purulent secretion in the openings of bronchi in some cases. Bronchial ulcer was also found in two patients with COVID-19 and IPA, as shown in Fig. 1c, d. However, a biopsy was not performed because of the severity of cases.

**Fig. 1:**
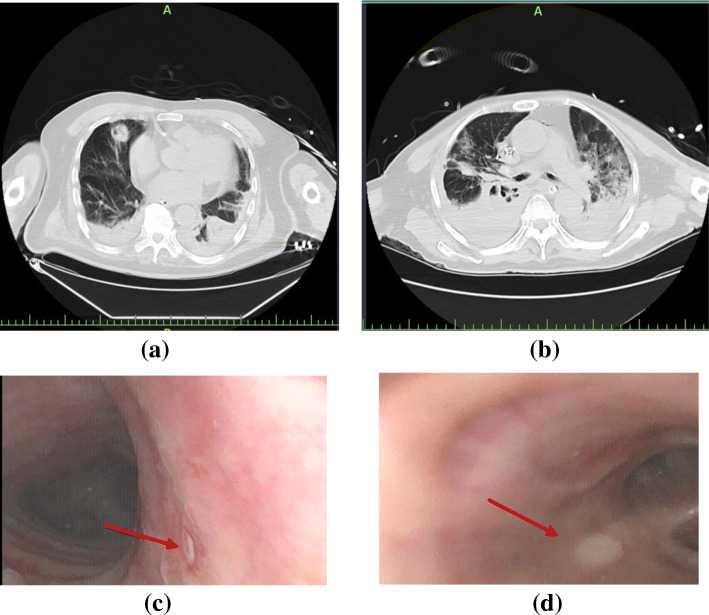
Lung CT scans and bronchoscopy findings of patients with COVID-2019 and IPA. Patient 1, 90 years, lung CT scan showed nodules with cavities in the middle lobe of the right lung and consolidation bilateral lower lobes with a small amount of pleural effusion (**a**); bronchoscopy finding showed small ulcer in the right wall of the trachea (**c**). Patient 2, 74 years, lung CT scan showed consolidation bilateral lower lobes with multiple irregular cavities in the middle (**b**); bronchoscopy finding showed right middle bronchial ulcer (**d**). Red arrow indicated the positions of bronchial ulcers

In summary, in our experience, the incidence rate of IPA among the patients with COVID-19 was obviously lower than those among patients with influenza (7.7% vs 19%) [4]. Older age, initial antibiotic usage of β-lactamase inhibitor combination, mechanical ventilation, and COPD were the risk factors of IPA among patients with COVID-19. Early intervention with bronchoscopy, observation of changes in the bronchial mucosa, and obtaining evidence of fungal microbiology were important in patients with severe/critical COVID-19.

## Data Availability

The datasets and materials used and/or analyzed during the current study are available from the corresponding author on reasonable request.
